# Development of an mRNA-LNP Vaccine against SARS-CoV-2: Evaluation of Immune Response in Mouse and Rhesus Macaque

**DOI:** 10.3390/vaccines9091007

**Published:** 2021-09-10

**Authors:** Alireza Naderi Sohi, Jafar Kiani, Ehsan Arefian, Arezou Khosrojerdi, Zahra Fekrirad, Shokoofeh Ghaemi, Mohammad Kazem Zim, Arsalan Jalili, Nazila Bostanshirin, Masoud Soleimani

**Affiliations:** 1 Celltech Pharmed Company, Tehran 1371616312, Iran; nanosohi@strc.ac.ir; 2 Department of Molecular Medicine, Faculty of Advanced Technologies in Medicine, Iran University of Medical Sciences, Tehran 1449614535, Iran; kiani.ja@iums.ac.ir; 3 Department of Microbiology, School of Biology, College of Science, University of Tehran, Tehran 1417935840, Iran; arefian@ut.ac.ir (E.A.); fekrirad@ut.ac.ir (Z.F.); shokoofeh.ghaemi@gmail.com (S.G.); 4 Department of Immunology, Faculty of Medical Sciences, Tarbiat Modares University, Tehran 1411713116, Iran; arezou.khosrojerdi@modares.ac.ir; 5 Department of Biotechnology, College of Science, University of Tehran, Tehran 1417935840, Iran; mk.zim73@ut.ac.ir; 6 Cell Science Research Center, Department of Stem Cells and Developmental Biology, Royan Institute for Stem Cell Biology and Technology, ACER, Tehran 16635-148, Iran; Jalili.arsalan@royaninstitute.org; 7 Hematopoietic Stem Cell Research Center, Shahid Beheshti University of Medical Sciences, Tehran 1983969411, Iran; 8 Department of Microbiology, School of Medicine Science, Alborz University of Medical Science, Karaj 3149779453, Iran; n.bostan@abzums.ac.ir; 9 Department of Hematology, Faculty of Medical Sciences, Tarbiat Modares University, Tehran 1411713116, Iran; 10 Department of Tissue Engineering and Applied Cell Sciences, School of Advanced Technologies in Medicine, Shahid Beheshti University of Medical Sciences, Tehran 1983969411, Iran

**Keywords:** mRNA-vaccine, lipid nanoparticle, LNP, SARS-CoV-2, spike protein

## Abstract

Among the vaccines have been developed thus far against SARS-CoV-2, the mRNA-based ones have demonstrated more promising results regarding both safety and efficacy. Two remarkable features of the mRNA vaccines introduced by the Pfizer/BioNTech and Moderna companies are the use of (N^1^-methyl-pseudouridine-) modified mRNA and the microfluidics-based production of lipid nanoparticles (LNPs) as the carrier. In the present study, except Anti-Reverse Cap Analog (ARCA), no other nucleoside analogs were employed to synthesize Spike-encoding mRNA using the in vitro transcription (IVT) method. Furthermore, LNPs were prepared via the ethanol injection method commonly used for liposome formation as an alternative for microfluidics-based approaches. The produced mRNA-LNP vaccine was evaluated for nanoparticles characteristics, encapsulation and transfection efficiencies, in vitro cytotoxicity as well as stability and storability. The safety of vaccine was assessed in Balb/c mice injected with mRNA-LNPs containing 10 µg of spike-encoding mRNA. Eventually, the vaccine efficacy in inducing an immune response against SARS-CoV-2 was studied in Balb/c and C57BL/6 mice (received either 1 or 10 µg of mRNA) as well as in rhesus macaque monkeys (infused with mRNA-LNPs containing 100 µg of mRNA). The ELISA and virus neutralizing test (VNT) results showed a significant augmentation in the level of neutralizing antibodies against SARS-CoV-2. Moreover, the ELISA assay showed virus-specific IFN-γ secretion in immunized mice as a marker of T_H_1 cell-based immune response, whereas favorably no change in the production of IL-4 was detected.

## 1. Introduction

Since the outbreak of COVID-19, various research programs have been launched to produce prophylactic vaccines, some of which have succeeded in completing clinical trials and entering the market. Generally, except the inactivated/attenuated virus vaccines, in other types of introduced or developing ones (e.g., non-replicating viral vector, protein subunit, and mRNA vaccines), the SARS-CoV-2 Spike glycoprotein (abbreviated as S-protein) has been considered as the main antigenic candidate for vaccine design and production [1,2]. It is well documented that SARS-CoV2 attaches to the human target cell through the interaction between the receptor binding domain (RBD) located in the Spike protein and its principal receptor, known as human Angiotensin Converting Enzyme 2 (hACE2), which is overexpressed in some organs, especially the lungs [3]. In fact, the Spike protein is a multifunctional virus component playing the crucial roles in preliminary virus–host cell binding, fusion, and eventually, cell entry [4]. Hence, the immunoglobulins produced against the Spike RBD region serve as the virus neutralizing antibodies [5]. Among the anti-SARS-CoV-2 vaccine platforms licensed by the regulatory organizations for public vaccination, the mRNA vaccines developed by the Pfizer-BioNTech (i.e., BNT162b) [6] and Moderna (i.e., mRNA-1273) [7] companies have demonstrated interesting prosperity in both efficacy and fewer side effects concerns. Some benefits over the production phase can be proposed for mRNA-based vaccines compared to other kinds. For instance, mRNAs can be rapidly produced using enzymatic in vitro translation (IVT) as a high throughput procedure within a few days of desired gene sequencing [8,9]. Since the chemical structures of mRNAs encoding different antigens resemble each other, the design and development of new mRNA vaccines needs similar steps [10]. In addition, even in mass production, manufacturing the mRNA vaccines follows an entirely virus- and cell-free procedure. Some mRNA modifications have been proposed to enhance its translation rate and the presentation of the resulting protein by the antigen presenting cells (APCs). Utilizing optimized 5′ cap and nucleoside analogs (e.g., N^1^-methyl-pseudouridine, m1Ψ) as well as the insertion of the RNA polymerase encoding region to obtain self-amplifying mRNA are two of the suggested approaches [11,12,13]. Due to some features such as appropriate biocompatibility, long-term stability, and high loading capacity, lipid nanoparticles (LNPs) are the most practical materials for mRNA encapsulation and delivery. Moreover, T-junction (mixer) microfluidic chips can be considered as the most prevalent tools for LNP preparation. In all T-mixer devices, regardless of channel geometry and architectures, the aqueous solution containing purified mRNA meets the lipid ingredients dissolved in ethanol at the junction, resulting in the spontaneous formation of mRNA encapsulating LNPs. The flow rate of each solution before being mixed at the junction is well tunable as a determinative factor in LNP properties including size and encapsulation efficiency [14]. Nevertheless, the microfluidic chips, especially the configurationally optimized ones, are basically costly consumables. Furthermore, precise accessories, such as peristaltic pumps, pressure driven pumps, or novel combined microfluidic instruments, are essential requirements [15]. In the present study, a novel mRNA-LNP vaccine was prepared and evaluated in Balb/c and C57BL/6 mice as well as in rhesus monkeys. Briefly, the following three highlights can be considered: (a) an unmodified Spike-encoding mRNA was utilized instead of nucleoside modified or self-amplifying ones; (b) LNPs were prepared through the ethanol injection method as a surrogate for microfluidics-based approaches; (c) the ionizable lipid employed herein was a commercially available lipid, unlike those applied by the Moderna and Pfizer/BioNTech companies.

## 2. Materials and Methods

### 2.1. Bioinformatics Studies and Vector Construction

The sequence of SARS-CoV-2 virus Spike gene was elicited regarding the valid databases (e.g., https://www.gisaid.org/, https://nextstrain.org/, https://www.ncbi.nlm.nih.gov/) (asscssed on 18 July 2020) and SARS-CoV-2 S-2P, comprising proline substitutions at residues K986 and V987, was chosen as the target antigen. GFP gene sequence was employed to produce a reporter mRNA model. The coding region of GFP and S protein, flanked by the optimized 5′- and 3′-UTRs and poly A tail, were designed and synthetized under the control of T7 bacteriophage promoter and the entire sequence was inserted within the PUC57 vector. Following the sequencing of the products and, consequently, the final approval, they were transformed into Stbl4 strain of *E. coli*, known to be useful for plasmid reception as well as enormous amplification of vectors.

### 2.2. In Vitro Transcription (IVT)

To perform IVT process, standard NEB mRNA Synthesis Kit (E2060) was used as described by the manufacturer. Necessarily, the concentrations of NTPs employed at IVT process as well as Ambion^®^ Anti-Reverse Cap Analog (ARCA, Thermofisher, Waltham, MA, USA) were optimized. No other nucleoside analog was employed instead of unmodified NTPs. The mRNA purification was carried out using the Monarch RNA cleanup kit (T2040L). Finally, the quantity of transcribed mRNA was measured using a nanodrop UV spectrophotometer (epoch2-Biotek, Winooski, VT, USA). Quality control was considered to determine the amount of host (i.e., *E. coli*) genomic DNA as well as protein impurities. Serving this purpose, PicoGreen^®^ dsDNA Quantitation Reagent was employed to detect DNA contamination. The likely protein impurities were also assessed using Pierce™ BCA Protein Assay Kit. The amount of bacterial endotoxin lipopolysaccharide (LPS) was also estimated through chromogenic limulus amebocyte lysate (LAL) assay.

### 2.3. Lipid Nanoparticle Production

Lipid nanoparticle (LNP) formulation was prepared as previously described for siRNA and mRNA delivery with remarkable modifications [16,17,18]. Briefly, lipids were dissolved in ethanol absolute at molar ratios of 50 (DLin-MC3-DMA, MedChenExpress): 10 (DSPC, Sigma, St. Louis, MI, USA): 38.5 (Cholesterol, Sigma): 1.5 (DMG-PEG_2000_, Sigma). mRNA was, in parallel, dissolved in sodium citrate buffer (50 mM, pH 4) within the RNase-free tube. After passing the solutions through a 0.22-micrometer filter, they were blended at the ratio of 3:1 (aqueous: ethanol) via ethanol injection method widely used for liposome production. The procedure was performed entirely under sterile conditions. After a 30-min interval, the obtained nanoparticles were diluted in sterile PBS (10 mM, pH 7.2) and transferred to pre-sterilized Amicon^®^ Ultra-15 centrifugal filters (cut off = 100 KDa). To implement buffer-exchange and product concentration, centrifugation was accomplished in 15–30 min at 4000× *g* and repeated three times after the addition of fresh PBS. The final product was stored at 4 ℃ until use.

### 2.4. Nanoparticle Characterization

Dynamic Light Scattering (DLS) was used to determine the size of mRNA-LNPs obtained from the previous step. For this purpose, the concentrated sample was diluted again with the ratio of 1:100 in sterile PBS. The test was carried out in triplicate at 25 ℃ and with a dispersion angle of 90° using a HORIBA-SZ100 device. To meticulously investigate possible aggregation of particles, the results were reported as the size versus intensity. Zeta potential was also measured in parallel.

### 2.5. Determination of Encapsulation Efficiency

Quant-iT™ RiboGreen™ RNA Assay kit was employed to calculate mRNA encapsulation efficiency as described by the manufacturer. Briefly, rRNA standard solutions with the different concentrations (i.e., 1000, 500, 250, 125, 62.5, 31.25, 15.625, and 0 ng/mL) were prepared in TE (Tris-EDTA) buffer. mRNA-LNPs provided from mixing the aqueous and ethanol solutions (as described in the previous section) before and after buffer exchange steps, were also eluted in TE to obtain a sample containing approximately 250 ng/mL mRNA. The similar samples were also made in TE buffer supplemented by Triton-X100 surfactant (0.5%). Into the microwells of a 96 well-plate, 100 µL of each sample (including standard solutions and mRNA-LNP samples) was poured. Afterwards, 100 µL of 1:200 diluted Ribogreen reagent was added. After a 5-min incubation in the dark and at room temperature, fluorescence intensity was recorded using Cytation 3 (Biotek, Winooski, VT, USA), applying the excitation and emission at 485 and 528 nm, respectively. The fluorescence obtained from mRNA-LNP samples dispersed in TE and TE/Triton-X100 was theoretically attributed to free (unencapsulated) and total mRNAs, respectively. Nevertheless, Ribogreen reagent can penetrate the LNPs slightly, even in the absence of Triton-X100. Hence, the fluorescence obtained from the filtered sample dispersed in TE was subtracted from that emitted from the similar unfiltered one in TE. A standard curve plotted using standard solutions was utilized to convert fluorescence intensity to the concentration. Finally, the encapsulation efficiency was calculated according to the following formula (in which “*W*” represents wight):

(1) $$\%\mathrm{EE}=\frac{W\left(total\;RNA\right)-W\left(free\;RNA\right)}{W\left(total\;RNA\right)}\times 100$$

### 2.6. In Vitro Transfection Efficiency and Expression

First, GFP-encoding mRNA-LNPs were applied to estimate in vitro transfection efficiency. Three different types of cells were engaged herein, including HEK 293T cells (as a cell line with a great propensity for transfection), Umbilical cord blood (UCB)-derived mesenchymal stem cells (as a human primary cell), and KG-1 (as a human-derived dendritic-like cell line). All cells were obtained from Iranian Biological Resource Center (IBRC). Both HEK 293T cells and UCB-derived mesenchymal stem cells were cultured in Dulbecco’s Modified Eagle’s Medium (DMEM) F-12, whereas suspension culture in RPMI1640 was performed for KG-1 cells. GFP-encoding mRNA-LNPs (0.5 µg of encapsulated mRNA/cm^2^) were added to the cells with about 70% confluency. Cells were observed using the fluorescence microscopy after 24–48 h. To further evaluate transfection efficiency as well as surface localization of expressed spike protein, KG-1 cells were treated by Spike-encoding mRNA-LNPs containing 0.5 or 1 µg of encapsulated mRNA/cm^2^. After 24 h cells were stained by CR3022 (human anti-SARS-CoV-2 Spike glycoprotein S1, Abcam, Waltham, MA, USA) antibody. Goat anti-human IgG H&L-FITC (Abcam, Waltham, MA, USA) was used as the secondary antibody. Then, the cells were investigated through flowcytometry, and the results were analyzed using FlowJo V10 software.

### 2.7. mRNA-LNP Stability and Storability

To evaluate the stability and shelf life of lipid nanoparticles (LNPs) containing mRNA, three parameters including size, zeta potential, and pH were redetermined after being stored for 30 days within refrigerator (5 ± 3 °C), followed by 0, 1, 2, 4, 24, and 48 h at room temperature (24 ± 2 °C). The possible leakage of encapsulated mRNA outside the nanoparticles was also investigated using Quant-iT™ RiboGreen™ RNA Assay kit as described. The ratio of fluorescence intensity obtained from the samples dispersed in TE to the ones dispersed in TE/Triton-X100 was employed as the determinant parameter.

### 2.8. In Vitro Cytotoxicity of mRNA-LNPs

To study the likely cytotoxicity of mRNA-LNPs, suspension cell culture and adherent cell culture of KG-1 and HEK 293T cell lines were performed, respectively. Briefly, after 24 h from cell seeding in 96 well-plates, different concentrations of mRNA-LNPs containing 0.25, 0.5, 1, or 2 µg of Spike-encoding mRNA were added. Cell viability was studied using an XTT assay (Abcam, Waltham, MA, USA) in two-time intervals of 24 and 72 h after mRNA-LNP addition.

### 2.9. Animal Studies

First, to evaluate synthetic Spike-encoding mRNA potency in inducing a humoral immune response, a single injection experiment using Lipofectamine (Thermofisher, Waltham, MA, USA) as the carrier was performed. Two groups of Balb/c mice were injected intramuscularly (I.M.) with 100 µL of Lipofectamine (diluted in sterile PBS) containing either 10 or 20 µg of Spike-encoding mRNA (eight mice in each mRNA receiving group and six mice in control group). The blood was collected on days 21, 28, and 84 post injection and analyzed following the serum separation. In the next step, vaccine efficiency was evaluated in the following two types of mice: Balb/c (6–8 weeks) and C57BL/6 (6–8 weeks) and ultimately, in rhesus macaque monkeys (4–6 years) as a non-human primates (NHPs) model. The mice were maintained under the standard conditions (25 °C and 12-h light/dark cycle) and nourished with the normal diet and adequate water. Mice of each species were randomly divided into two groups receiving either 1 or 10 µg of Spike-encoding mRNA (eight mice in each group). Another group (including eight mice) randomly selected from Balb/c mice were employed as the control to receive PBS only. The blood samples were gathered after two weeks (14 days). In the following, the second (i.e., booster) injection was fulfilled after three weeks (i.e., 21 days after the first infusion). Two weeks later (i.e., 35 days after the initial injection) the blood samples were collected to be analyzed. To evaluate the efficacy of the vaccine in rhesus monkeys, 1 mL of mRNA-LNP solution containing 100 µg of Spike-encoding mRNA was intramuscularly (I.M.) injected into the three monkeys. As the control negative sample, two monkeys received the same volume of PBS only. The second injection was performed 28 days (four weeks) after the first one following the blood sampling. The blood samples were recollected four weeks after the second inoculation (day 56 post first injection). The study was conducted according to the guidelines of the Declaration of Helsinki, and approved by the research ethics committee of Iran Ministry of Health and Medical Education (Approval ID: IR.SBMU.RETECH.REC.1399.839, Approval Date: 29 December 2020).

### 2.10. Investigation of Humoral Immune Response

To survey the humoral immune response generated by administration of Spike-encoding mRNA-LNPs, the indirect configuration of enzyme-linked immunosorbent assay (ELISA) method was used. Briefly, the gathered bloods were allowed to clot at room temperature in serum separator tubes. Subsequently, the tubes were centrifuged at 5000 g for 10 min and the sera samples were collected meticulously. SARS-CoV-2 RBD protein coated 96 well-microplate (Ray Biotech, Atlanta, GA, USA) was employed. Sera were diluted 1:1000 with the diluent buffer prepared and optimized in our laboratory. HRP goat anti-mouse IgG (minimal x-reactivity) antibody (BioLegend, San Diego, CA, USA) and HRP goat anti-monkey IgG H&L antibody (Abcam, Waltham, MA, USA) were used as the secondary antibodies to detect anti-Spike RBD IgG antibodies in sera of mice and monkeys, respectively. TMB/H_2_O_2_ was utilized as the chromogenic substrate. Optical density (OD) was measured at 450 nm using an Epoch 2 microplate spectrophotometer (Biotek, Winooski, VT, USA). All sera were analyzed in duplicate. To determine reciprocal endpoint titer, serial dilutions of monkeys’ sera 56 days after the first injection of mRNA-LNPs (containing 100 µg Spike encoding mRNA) were prepared from 1:100 up to 1:10,000. The last dilution in which the obtained OD was above the cut off index was recorded.

### 2.11. Virus Neutralization Test (VNT)

To further analyze the humoral immune response induced by mRNA-LNPs, virus neutralization test was performed in Amirabad Virology Laboratory (Tehran, Iran), as described previously [19]. Briefly, twofold serial dilutions of C57BL/6 and Balb/c mice sera were prepared in 2% DMEM. The sera were obtained from the mice that received 10 µg of Spike-encoding mRNA, two weeks after the second injection. Afterwards, 100 TCID_50_ of SARS-CoV-2 (Wuhan strain isolated from infected patients) was added and a 60-min incubation at 37 ℃ was conducted. The mixtures were added to VeroE6 cells and incubated at 37 ℃ and 5% CO_2_ atmosphere. Finally, the cytopathic effect (CPE) was investigated and scored.

### 2.12. Investigation of Cell-Mediated Immune Response

To evaluate cell-mediated immune response, spleen tissues were collected from Balb/c mice that received mRNA-LNPs containing either 1 or 10 µg of Spike encoding mRNA at day 28 post booster immunization. Mouse uncoated ELISA kits (Invitrogen, Waltham, MA, USA) were used upon stimulation of elicited cells with peptide pool comprising SARS-CoV-2 Spike RBD to quantify interferon γ (IFN-γ) and interleukin-4 (IL-4) secretions. Cell activation cocktail (without Brefeldin A, BioLegend, San Diego, CA, USA) and RPMI 1640 medium were employed as the positive and negative controls, respectively.

### 2.13. Statistical Analysis

All the obtained information was evaluated using GraphPad Prism 9 software. *t*-test and one-way ANOVA (followed by Tukey’s post-test) algorithms were used as required to analyze the results statistically. A *p*-value lower than 0.5 was considered as the significant difference.

## 3. Results

### 3.1. IVT Reaction Optimization

For the IVT to have the highest efficiency, various concentrations of NTPs and different incubation times were examined (data not shown). Finally, the optimal concentration of ATP, CTP, and UTP was obtained as 4 mM. The best concentrations of GTP and ARCA were also obtained as 2 and 8 mM, respectively. Furthermore, a 3-h incubation was determined as the optimized time for the enzymatic reaction to be completed.

### 3.2. mRNA Purity

Probable *E. coli*-originated DNA contamination as well as protein impurities in the transcripted mRNA were surveyed using PicoGreen and BCA kits, respectively. Based on the results, the DNA and protein impurities were in an acceptable range of <0.01 and <5.0 µg/mL, respectively. In addition, the endotoxins level was <0.4 EU/mL and passable. The level of endotoxins was re-examined after the LNP formation, before and after storing. No significant change was recorded.

### 3.3. mRNA-LNP Size and Zeta Potential

**Figure 1:**
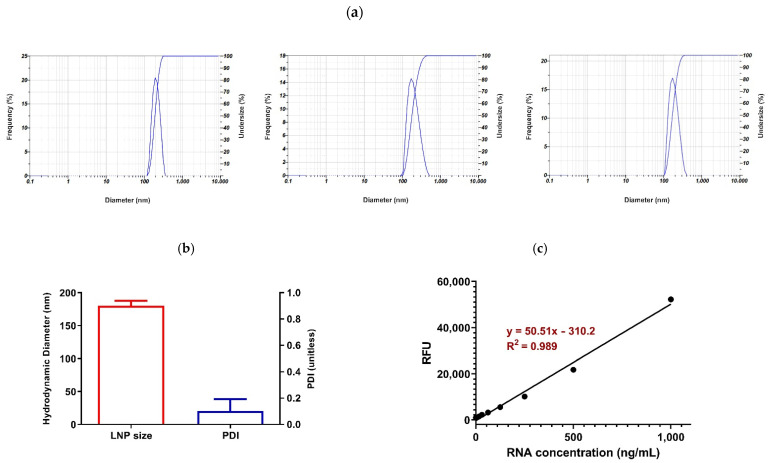
Characterization of lipid nanoparticles (LNPs) obtained using ethanol injection method. (**a**) DLS results representing hydrodynamic diameter vs. Intensity. (**b**) Average of hydrodynamic diameter ± SD and average of polydispersity index (PDI) ± SD. (**c**) Standard curve showing linear relationship between RNA concentration and fluorescence intensity.

According to the results obtained using DLS, the hydrodynamic diameter of the nanoparticles was 180.06 ± 7.89 nm (CV% = 4.38%). Moreover, the polydispersity index (PDI) showed an acceptable value (0.104 ± 0.089), indicating the size uniformity of the prepared mRNA-LNPs (Figure 1a,b). A singlet peak appearing in the DLS results, especially reported by the intensity, implies the absence of any nanoparticles aggregation. The zeta potential was also measured as −3.5 ± 2.12.

### 3.4. Encapsulation Efficiency (% EE)

To evaluate the efficiency of lipid nanoparticles in mRNA encapsulation, a Quant-iT™ RiboGreen™ RNA Assay kit was employed, as aforementioned. The standard curve represented the linear relationship (R^2^ = 0.989) between the mRNA concentration and the fluorescence intensity, which was utilized for the estimation of the encapsulation efficiency, obtained as about 95% (Figure 1c).

### 3.5. In Vitro Transfection Efficiency and Cell Surface Localization

**Figure 2:**
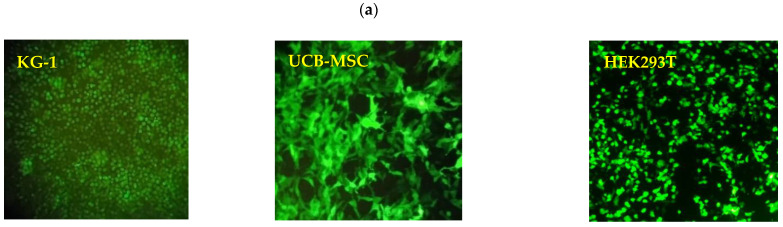

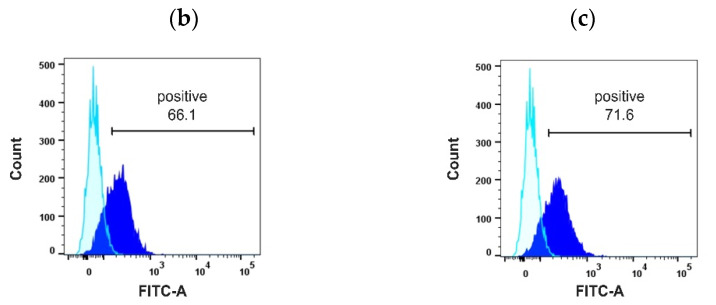
(**a**) Investigating transfection efficiency of GFP-encoding mRNA-LNPs in three types of cells including KG-1, UCB-mesenchymal stem cells, and HEK293 using fluorescence microscopy. (**b**,**c**) Determination of surface localization of translated Spike protein in KG-1 using flowcytometry analysis when LNPs containing 0.5 and 1 µg of Spike-encoding mRNAs/cm^2^ were used, respectively.

Since the mRNA vaccine was designed to target antigen-presenting cells, to evaluate the mRNA-LNPs regarding cell entry capability, endosomal escape, cargo release, and ultimately, the translation of the transported mRNA molecules, the expression of green fluorescence protein (GFP) was studied in three different types of cells (HEK 293T, KG-1, and Umbilical cord blood (UCB)-derived mesenchymal stem cells) after exposure to GFP-encoding mRNA-LNPs. The results obtained using fluorescence microscopy demonstrated 90–100% of the cell population expressing GFP in all the three types of cells studied (Figure 2a). It indicated the prosperity of mRNA-LNPs to overcome all the probabilistic barriers toward the delivery and translation of encapsulated mRNA. Moreover, considering the flowcytometry results, 66.1% and 71.6% of KG-1 cells represented the cell surface localization of the Spike protein when they were treated by LNPs encapsulating 0.5 and 1 µg of Spike-encoding mRNAs/cm^2^, respectively (Figure 2b,c).

### 3.6. mRNA-LNP Stability and Storability

**Figure 3:**
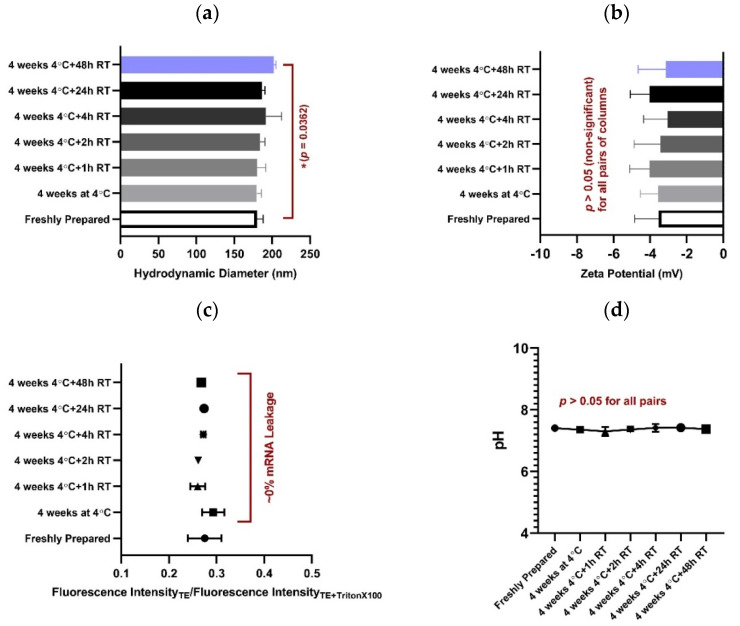
Evaluating mRNA-LNP stability and storability. (**a**) No significant change in nanoparticles size was observed in any of the groups comparing to the control (freshly prepared) one when data were analyzed using One-way ANOVA (*p* > 0.05). By employing unpaired *t*-test, a significant increase in size of LNPs kept at room temperature for 48 h following one month being refrigerated was deduced (*p* = 0.0362). Nevertheless, no significant change in zeta potential (**b**), no mRNA leakage (**c**), and no change in pH (**d**) was detected in any of the groups compared to the control one.

To investigate the change in size and zeta potential of nanoparticles, DLS and Zeta sizer were used. The results showed no significant change in the size of nanoparticles compared to the control (freshly prepared) sample when the results were analyzed statistically using a one-way ANOVA (Figure 3a). Nevertheless, by employing an unpaired *t*-test, the sample kept at room temperature for 48 h following one month of being refrigerated showed a significant increase in size compared to the control one (*p* = 0.0362). No alterations were observed in the zeta potential in any of the cases (Figure 3b). Moreover, no mRNA leakage from the nanoparticles was detected (Figure 3c). The pH of all the samples remained as 7.3–7.4 due to the buffering performance of PBS (Figure 3d).

### 3.7. In Vitro Cytotoxicity Evaluation of mRNA-LNPs

**Figure 4:**
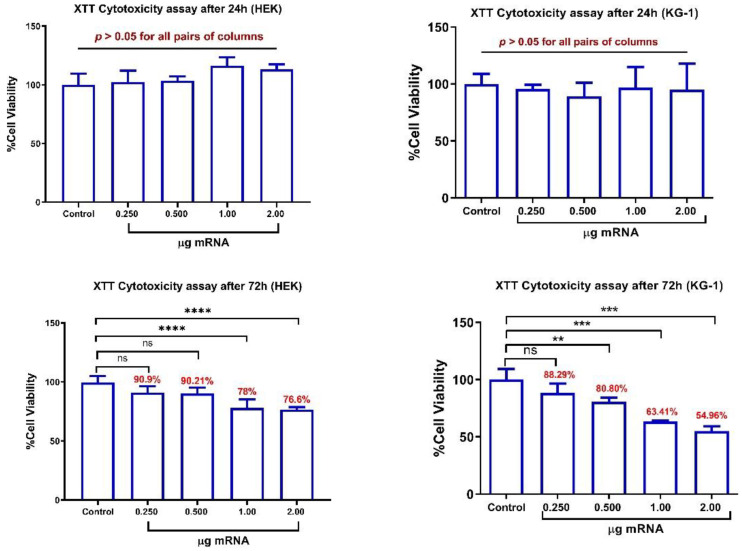
Cytotoxicity evaluation of mRNA-LNPs using XTT viability assay. HEK293T and KG-1 cells were treated by different amounts of Spike-encoding mRNA-LNPs for 24 and 72 h. ns: non-significant, ** *p* < 0.01, *** *p* < 0.001, **** *p* < 0.0001.

The results showed no significant change in the viability of both studied cell lines (i.e., KG-1 and HEK293T) after 24 h of continuous treatment by mRNA-LNPs. However, after 72 h, KG-1 cells showed a significant decrease in viability when they were treated by mRNA-LNPs containing ≥ 0.5 µg mRNA (equivalent to ≥1.56 µg of mRNA/cm^2^). Likewise, the viability of HEK293T cells was decreased significantly after a 72-h treatment by the nanoparticles containing ≥ 1.0 µg of mRNA (equivalent to ≥3.12 µg of mRNA/cm^2^). Nevertheless, even after 72 h of continuous treatment by the highest quantities of mRNA-LNPs containing 2 µg of encapsulated mRNA, the viability remained above 54 and 76% in KG-1 and HEK293T cell lines, respectively (Figure 4).

### 3.8. Vaccine Safety

**Figure 5:**
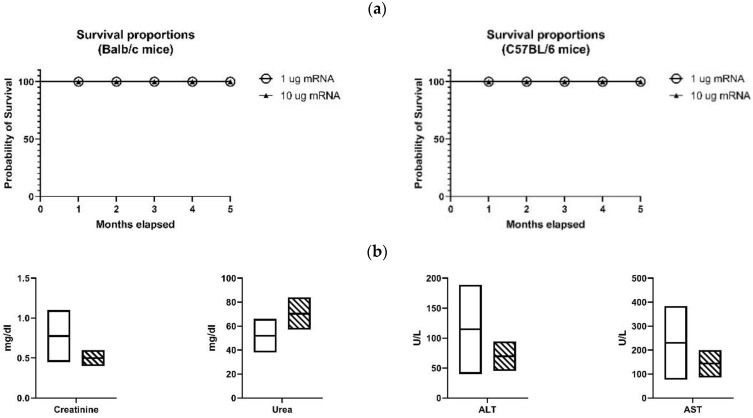
Evaluating vaccine safety in mice. (**a**) Survival proportions of Balb/c and C57BL/6 mice received mRNA-LNP vaccine containing either 1 or 10 µg of Spike-encoding mRNA. No death was recorded after 5 months of monitoring. (**b**) The serum level of Cretinine, Urea, ALT, and AST in Balb/c mice that received mRNA-LNP vaccine containing 10 µg of Spike-encoding mRNA one week after the 2nd injection. The crosshatched and white rectangles show the samples results and normal ranges, respectively.

Five C57BL/6 and five Balb/C mice received the mRNA-LNP vaccine containing 1 µg of Spike-encoding mRNA. In a similar grouping, the mice were inoculated with the mRNA-LNP vaccine containing 10 µg of Spike-encoding mRNA. All the groups received the booster injection after 4 weeks. Favorably, no deaths were observed after 5 months in any of the groups (Figure 5a). Additionally, the levels of creatinine, urea, ALT (SGPT), and AST (SGOT) in the sera of the five Balb/c mice that received 10 µg of Spike-encoding mRNA were analyzed one week after the second infusion (Figure 5b). The creatinine serum level, as the most important parameter for evaluating the renal function, was in the normal range; however, the level of urea was elevated in two cases, often referring to body dehydration as a common reversible phenomenon. The levels of hepatic enzymes, ALT and AST, as the main blood factors to investigate the liver health were in the normal range as well.

### 3.9. Vaccine Immunogenicity in Mice

**Figure 6:**
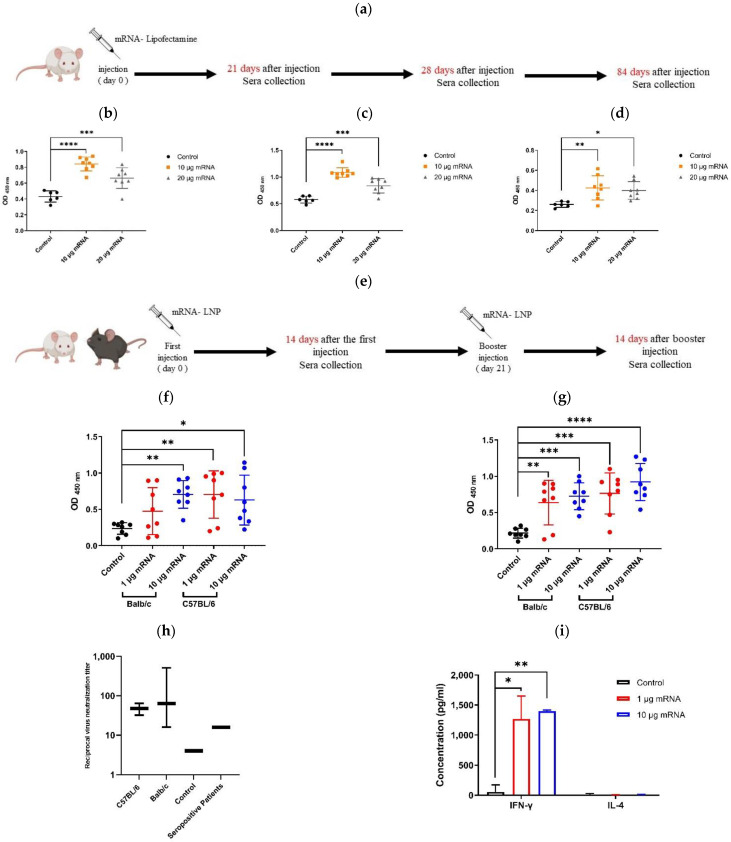
Investigating vaccine immunogenicity in mice. (**a**) Schedule of sera collection after injection of Spike-encoding mRNA-Lipofectamine mixture. ELISA results showing comparison between serum level of anti-Spike RBD (neutralizing) antibodies in Balb/c mice that received mRNA–Lipofectamine mixture containing either 10 or 20 µg of mRNA and the control group at days 21 (**b**), 28 (**c**), and 84 (**d**) after injection. (**e**) Schedule of mRNA-LNP injection into Balb/c and C57BL/6 mice and sera collection. ELISA results showing comparison between serum level of anti-Spike RBD (neutralizing) antibodies in Balb/c and C57BL/6 mice that received mRNA-LNPs containing either 1 or 10 µg of mRNA and the control group at days 14 (**f**) and 35 (**g**) after the 1st immunization. (**h**) Virus neutralization test (VNT) showing reciprocal virus neutralization titer of Balb/c and C57BL/6 mice sera received mRNA-LNPs containing 10 µg of Spike-encoding mRNA and comparison to the control group as well as two seropositive patients. (**i**) Secretion of IFN-γ and IL-4 in splenocytes stimulated with the SARS-CoV-2 RBD measured using an ELISA assay. * *p* < 0.05, ** *p* < 0.01, *** *p* < 0.001, **** *p* < 0.0001.

As aforementioned, the efficiency of the prepared Spike-encoding mRNA in inducing a humoral immune response was first investigated using Lipofectamine as the transporter (Figure 6a). The results demonstrated a significant rise in the anti-spike RBD IgG antibodies in the mice sera that received mRNA, compared to the control group in all the examined days (i.e., days 21, 28, and 84) (Figure 6b–d). Interestingly, the injection of 10 µg of mRNA was more favored than the 20 µg one in augmentation of the anti-Spike antibodies level. Despite not being injected with a second dose, the higher level of serum antibodies retained compared to the control group during the study period (up to 84 days) was quite promising. In the following, to evaluate the immunogenicity of the final formula of the vaccine, a two-dose mRNA-LNP vaccine containing either 1 or 10 µg of Spike-encoding mRNA was injected into two mice species, Balb/c and C57BL/6 (Figure 6e). The serologic results obtained at days 14 (two weeks after the initial injection) and 35 (two weeks after the second injection) confirmed a significant increase in the anti-Spike RBD IgG antibodies in all the groups that received the vaccine (Figure 6f,g). In the C57BL/6 mice, the first dose vaccination by LNPs containing 1 µg of mRNA was more efficacious than those with 10 µg of mRNA. However, after the booster dose, the vaccines containing 10 µg of mRNA showed further output in producing anti-Spike antibodies in both mice species compared to the control group. To investigate the efficacy of the generated antibodies in inhibiting the infectious function of SARS-CoV-2, a virus neutralization assay was performed (Figure 6h). The results indicate the potency of vaccinated Balb/c and C57BL/6 sera in virus neutralization when they were diluted up to 1:512 and 1:64, respectively. Finally, the ELISA results showed that the secretion of interferon γ (IFN-γ) in splenocytes elicited from the Balb/c mice that were vaccinated with either 1 or 10 µg of Spike-encoding mRNA was significantly further than in the control group (*p* < 0.05 and *p* < 0.01, respectively). Moreover, no significant difference in the secretion of IL-4 was detected between the vaccinated groups and the control one (Figure 6i).

### 3.10. Vaccine Immunogenicity in Rhesus Monkeys

**Figure 7:**
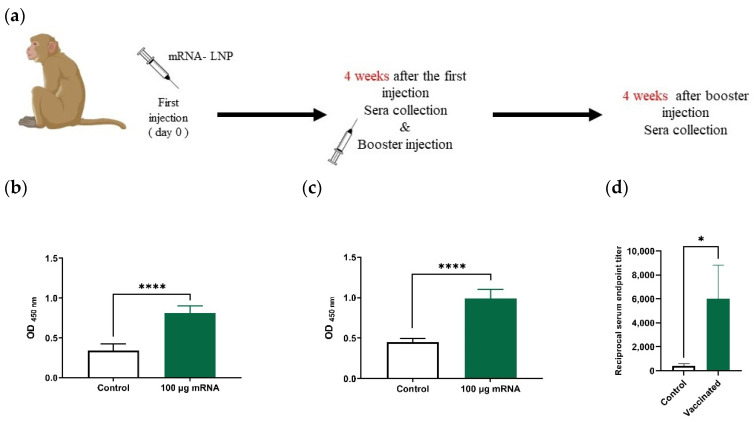
Investigating vaccine immunogenicity in rhesus macaque monkeys. (**a**) Schedule of mRNA-LNP injection into rhesus monkeys and sera collection. ELISA results showing comparison between serum level of anti-Spike RBD (neutralizing) antibodies in monkeys that received mRNA-LNP vaccine and the control group at days 28 (**b**), and 56 (**c**) after the 1st injection. (**d**) Reciprocal endpoint titer of monkey’s sera obtained by ELISA method. * *p* < 0.05, **** *p* < 0.0001.

Based on the serologic results obtained 28 and 56 days after the first injection (Figure 7a), a significant increase in the level of the anti-Spike RBD IgG antibody was observed in the sera of monkeys that received the mRNA-LNP vaccine compared to the control group (*p* < 0.0001) (Figure 7b,c). A reciprocal end point titer (Figure 7d) was also measured, demonstrating a significant difference between the vaccinated monkeys and the control one (*p* = 0.0333).

## 4. Discussion

mRNA vaccines have been introduced as extremely promising therapeutics after the COVID-19 pandemic. Dramatically, two of them developed by BioNTech/Pfizer and Moderna received a lot of attention after being highly effective in clinical trials followed by being used in broad public vaccination around the world. mRNA vaccines have some advantages compared to those that are DNA-based. As the most prominent feature, mRNAs would be delivered to the cytoplasm rather than the nucleus, making the risk of genomic integration implausible. The cell-free manufacturing of mRNA-vaccines is another distinctive characteristic of mRNA vaccines compared to DNA-based and inactivated virus vaccines. In the present study, a novel mRNA-LNP vaccine against SARS-CoV-2 was developed and preclinically evaluated. The mRNA used in this vaccine encodes the whole sequence of the Spike protein containing N and C-terminal domains. It is well documented that the S1 C-terminal domain (CTD) acts as the receptor binding domain (RBD) in SARS-CoV as well as SARS-CoV-2 [3]. The induction of an immune response against Spike RBD would serve evidently as a virus neutralizing reaction. Except for employing ARCA as the cap, the synthesis of mRNA through the enzymatic in vitro transcription (IVT) procedure was entirely accomplished using unmodified nucleoside triphosphates (NTPs). Among the carriers developed for drug and gene delivery, lipid nanoparticles (LNPs) are of great interest for the delivery of nucleic acid-based cargos, especially when the long-term stability and shelf-life as well as the minimal toxicity are considered. Hence, LNPs have been the first choice in the preparation of mRNA prophylactic (preventative) vaccines. However, for producing therapeutic ones (e.g., cancer vaccines), other options, such as liposome-based structures (e.g., lipoplex) with the lower storability and higher toxicity, would be acceptable [20]. In the present study, LNPs composed of lipid components formerly used for siRNA and mRNA delivery were employed for encapsulating and delivering a Spike-encoding mRNA to antigen presenting cells [16,17,18]. Herein, we utilized dilinoleylmethyl-4-dimethylaminobutyrate (with the acronym: D-Lin-MC3-DMA) as a commercially available ionizable lipid, whereas the BioNTech and Moderna companies used Heptadecan-9-yl 8-((2-hydroxyethyl) (6-oxo-6-(undecyloxy) hexyl) amino) octanoate (with the acronym: SM102) and ((4-hydroxybutyl) azanediyl) di (hexane-6,1-diyl) bis (2-hexyldecanoate) (with the acronym: ALC-0315) [21,22], respectively. Nevertheless, unlike the conventional use of microfluidics-based devices, which are widely considered for LNP formation, we employed the ethanol injection method as an uncomplicated approach frequently used to produce liposomes. It is worth noting that there are no specific guidelines approved by the FDA or European Medicines Agency (EMA) for the production of mRNA-based vaccines [23]. Even though the ethanol injection method lacks the tunable parameters involved in the microfluidics-based ones (e.g., flow rate, channel diameter, architecture, etc.), its simplicity and great reproducibility were highly remarkable. The most important advantage of using microfluidics technology is the ability to produce LNPs with a size below 100 nm, making their 0.22-micrometer filtration feasible. However, smaller mRNA-LNPs do not guarantee more immunogenicity. Contrarily, some previous studies revealed that larger mRNA-LNPs may be more successful in inducing an immune response [24]. Herein, we prepared mRNA-lipid nanoparticles with a diameter of about 180 nm and a narrow size distribution (PDI = 0.104). Since the sterile filtration of the end-product would be impossible, implementation of the robust sterile conditions in the upstream processes was required. Although there are disputed opinions in the literature about the effect of LNP size on its entrapping capability as well as cell entry, our LNPs demonstrated interesting encapsulation and transfection efficiencies (even in the KG-1 cell line with a low transfection rate) as well. The observed cell surface localization was in agreement to what was formerly reported when either a wild-type or 2P mutant spike encoding mRNA was used [25]. The stability and accelerated shelf-life of mRNA-LNP vaccines were examined after one month of being refrigerated. The results showed that mRNA-LNPs were stable even after 24 h at room temperature, making the vaccines favored for transportation and the likely negligence in maintenance before injection. This result agreed with the previous findings on the stability of LNPs with similar lipid ingredients [11]. According to the XTT viability assay, no significant cytotoxicity in either of the examined cell lines was observed, even after 72 h of continuous treatment by LNPs containing 0.25 µg of mRNA (equivalent to 0.78 µg of mRNA/cm^2^). The diminished viability of the cells after 72 h of exposure to the larger amounts of mRNA-LNPs could be attributed to cellular GTP depletion due to the overactivity of the translation machinery. Moreover, since the N/P ratio was constant, the higher level of presented mRNA was equal to the increase in the amounts of lipid components, probably above their biocompatible concentrations. In all the animal studies, the vaccine was well tolerated and no local adverse reaction, including inflammation or purpura, was detected after the mRNA-LNP injection. Moreover, the survival percentage of the mice that received either 1 or 10 µg of encapsulated mRNA was 100% up to five months after being inoculated. The inevitable entrance of the intramuscularly injected LNPs into systemic circulation leads to hepatic accumulation, particularly through opsonization by apolipoprotein E (Apo E) and then hepatocellular uptake by low-density lipoprotein receptor [23,26]. The sustained level of hepatic enzymes (i.e., ALT and AST) in the examined mice was in favor of liver non-toxicity. The efficiency of in vitro transcripted mRNA in inducing a humoral immune response was studied first using lipofectamine as a conventional in vitro transfecting reagent. Lipofectamine contains DOSPA (2,3-dioleoyloxy-N-[2(sperminecarboxamido)ethyl]-N,N-dimethyl-1-propaniminium trifluoroacetate) as a cationic lipid and DOPE (1,2-Dioleoyl-sn-glycero-3-phosphoethanolamine) as a helper lipid. The novel versions of lipofectamine (e.g., Invivofectamine, Thermofisher) are commercially available for the in vivo delivery of nucleic acids. However, due to the likely toxicity of cationic lipids as well as the instability of obtained lipoplexes, their application in prophylactic medicines is not recommended. Nevertheless, our results indicate acceptable permanency in the level of generated anti-Spike IgG antibodies after the one-dose injection of mRNA-Lipofectamine mixture to the Balb/c mice. This finding was highly remarkable regarding the fact that the use of unmodified mRNA agreed with the former studies, confirming the competence of unmodified mRNAs in vaccines’ development [27]. Eventually, the efficiency of the mRNA-LNP final formulation was evaluated in mice and rhesus monkeys as well. According to the serologic results, including ELISA and VNT assays, our unmodified mRNA-LNP vaccine had a significant potency in inducing a humoral immune response against SARS-CoV-2 in two species of mice as well as rhesus macaque monkeys as a non-human primate model. Given that Spike RBD protein was used as the coated antigen, the ELISA test actually determined the level of virus-neutralizing IgG antibodies [5]. This finding was further confirmed by a virus neutralization test on mice sera in which the serum of the vaccinated Balb/c, even with the dilution up to 1:512, was able to impede the virus-induced cytopathic effect in Vero-E6 cells via neutralizing SARS-CoV-2. RBD-stimulated splenocytes from Balb/c mice were employed to investigate the T_H_1 and T_H_2 cell-based immune responses through measuring IFN-γ and IL-4 secretions, respectively. Whereas the IFN-γ secretion was increased significantly in the vaccinated mice, no significant change in the level of IL-4 was observed. Since the T_H_2-biased immune response may result in vaccine-associated enhanced respiratory disease (VAERD) occurring as an unwanted consequence of vaccination [28,29], the absence of it after immunization with mRNA-LNPs was most significant.

## 5. Conclusions

This vaccine was manufactured from unmodified spike encoding mRNAs encapsulated within the lipid nanoparticles that were formed through the ethanol injection method. To the best of our knowledge, it was the first attempt to develop an mRNA vaccine against SARS-CoV-2 without using microfluidics or T-mixer-based equipment. Despite employing straightforward production approaches, it was concluded that this mRNA-LNP vaccine was efficient enough to induce high level production of neutralizing antibodies against SARS-CoV-2 in mice and rhesus macaque monkeys. Moreover, the IFN-γ assay demonstrated a significant induction of Spike RBD-specific T_H_1-cell response in the immunized mice. Further studies on safety and cellular immunogenicity in vaccinated non-human primates are ongoing in our lab.

## Data Availability

Not applicable.
